# Carbon Monoxide as a Cancer-Selective Anti-Neoplastic Agent in Esophageal Epithelial Cells

**DOI:** 10.3390/biom16101453

**Published:** 2026-10-05

**Authors:** Kinga Krukowska, Małgorzata Lasota, Monika Pinkas, Marcin Magierowski

**Affiliations:** 1 Center of Biomedicine and Interdisciplinary Sciences, Jagiellonian University Medical College, 16 Grzegórzecka Street, 31-531 Krakow, Poland; kinga.krukowska@doctoral.uj.edu.pl (K.K.); malgorzata.lasota@uj.edu.pl (M.L.); monika.pinkas@uj.edu.pl (M.P.); 2 Doctoral School of Medical and Health Sciences, Jagiellonian University Medical College, 16 św. Łazarza Street, 31-530 Kraków, Poland; 3 Department of Pharmacobiology, Jagiellonian University Medical College, 9 Medyczna Street, 30-688 Kraków, Poland

**Keywords:** esophageal adenocarcinoma, esophageal epithelium, carbon monoxide

## Abstract

Barrett’s esophagus (BE) is a premalignant adaptation to chronic gastroesophageal reflux disease (GERD) and the principal precursor lesion of esophageal adenocarcinoma (EAC). Carbon monoxide (CO), an endogenous gaseous mediator generated by heme oxygenases, regulates cellular homeostasis, stress responses, and survival pathways; however, its role in BE progression and EAC remains poorly understood. We investigated the biological and transcriptional effects of increased CO bioavailability in cellular models representing distinct stages of disease progression. Non-transformed esophageal squamous epithelial cells (NES-G2T), BE cells (BAR-T), and EAC-derived OE19 and OE33 cells were exposed to the CO donor CORM-A1 or its CO-depleted structural control (iCORM-A1). Cellular metabolic activity was assessed by the MTT assay, apoptosis by Annexin V/PI flow cytometry, and transcriptomic alterations by RNA sequencing in BAR-T, OE19, and OE33 cells following CORM-A1 treatment. Transient CO exposure was well tolerated by NES-G2T cells, with no significant effects on metabolic activity or apoptosis. While BAR-T cells showed alterations in MTT-derived metabolic activity, they exhibited only limited apoptotic changes following CORM-A1 exposure, indicating that the reduced metabolic activity was not accompanied by significant apoptotic cell death. In contrast, CORM-A1 induced a reduction in metabolic activity and increased apoptotic cell death in EAC-derived OE19 and OE33 cells. At 500 μM, CORM-A1 reduced metabolic activity of both EAC cell lines below 75% of the untreated control and exerted a significantly greater reduction in metabolic activity than iCORM-A1, suggesting a contribution of CO release to the observed response. Flow cytometric analyses confirmed a noticeable apoptotic response to CORM-A1 in both EAC cell lines. RNA sequencing revealed marked, highly cell type-dependent transcriptional responses to CO exposure. Non-neoplastic BAR-T cells exhibited the most pronounced transcriptomic remodeling, predominantly affecting pathways related to epithelial differentiation, tissue homeostasis, and extracellular matrix organization. In contrast, OE33 and OE19 cells displayed more restricted transcriptional responses that preferentially involved growth factor signaling, cellular adaptation, inflammatory regulation, and tumor-microenvironment interactions. Collectively, these findings suggest that responsiveness to CO is shaped by the degree of neoplastic transformation, with BE mounting a broad adaptive transcriptional response, showing limited drug-induced apoptosis. However, malignant cells exhibit greater vulnerability to CORM-A1-induced programmed cell death despite undergoing more limited transcriptomic remodeling. These data identify CORM-A1-responsive pathways and CO signaling as potential therapeutic targets in EAC.

## 1. Introduction

Esophageal adenocarcinoma (EAC) develops over decades through a multifactorial process involving chronic inflammation, epithelial remodeling, and progressive molecular alterations [1,2,3]. It follows a sequence of genetic and epigenetic alterations, beginning with gastroesophageal reflux disease (GERD), progressing through Barrett’s esophagus (BE), and subsequently accumulating dysplastic changes (low-grade and high-grade dysplasia) that ultimately culminate in invasive EAC [2,4,5]. GERD affects approximately 20% of society in Western populations [6]. The normal/physiological squamous epithelium (SE) of esophageal mucosa, when repeatedly exposed to gastric contents, acid, and bile, undergoes chronic cytokine-mediated inflammation and injury [3,7,8]. During a wound-healing regenerative process [9], the reflux-damaged squamous epithelium at the gastroesophageal junction is replaced by a columnar intestinal-type epithelium known as Barrett’s metaplasia or specialized intestinal metaplasia (SIM) [9,10,11]. The prevalence of this pre-malignant condition ranges from 0.8% to 23% in the general population, depending on risk factors, geographic region, confirmed GERD or BE family history [12]. It is estimated that fewer than 0.5% of BE patients progress to EAC [13]. The EAC prevalence is constantly growing [14,15]. Over the past few years, a growing body of evidence has highlighted the potential of molecular biomarkers in BE and EAC to improve diagnosis, prognostic stratification and predictive value [16,17,18]. Although biomarkers such as PD-L1 and HER2 expression, as well as mismatch repair deficiency and microsatellite instability status, are established in the clinical management of selected EAC patients, emerging molecular alterations, microRNAs, and circulating nucleic acids are increasingly being investigated as candidate diagnostic, prognostic, and predictive biomarkers [19]. Recent omics-based studies have identified numerous molecular biomarkers associated with BE and EAC development [20,21,22,23]. Several mechanistic hypotheses have been proposed to explain BE and EAC initiation and progression [9]. Despite substantial progress, the molecular mechanisms underlying BE and EAC development remain incompletely understood, and effective mechanism-based therapies are still lacking [24].

In parallel, three endogenous gaseous signaling molecules (gasotransmitters), namely nitric oxide (NO), hydrogen sulfide (H_2_S), and carbon monoxide (CO), have been identified and shown to exert pleiotropic biological activities, including within the gastrointestinal (GI) tract. Our recent work demonstrated that pathology-driven disruption of sulfide signaling is a key driver of redox remodeling and Barrett’s metaplasia development [25]. Despite its well-known toxicity at high concentrations [26], CO is also an endogenously produced signaling molecule generated during heme degradation by heme oxygenases (HMOXs). Owing to its high membrane permeability and affinity for transition metals, particularly heme-bound iron, CO participates in multiple physiological signaling pathways [27]. At low concentrations, CO exerts cytoprotective, regenerative, anti-inflammatory, and pro-resolving effects, suggesting therapeutic potential in gastric and esophageal mucosal disorders [28]. Both pro-tumorigenic and anti-tumorigenic effects of CO have been reported [29,30]. Additionally, CO can be delivered exogenously via metal-based CO-releasing molecules (CORMs) [31], organic, or esterase-sensitive organic CO-prodrugs [32,33], that release CO, also in a controllable manner via a click-and-release mechanism. CO-prodrugs were reported to accelerate the healing of experimental preexisting gastric ulcers [28], to reduce gastric toxicity of the commonly used non-steroidal anti-inflammatory drugs (e.g., aspirin), to counteract acute esophagitis in physiological esophageal epithelium or to reduce detrimental aspects of metabolic syndrome [34,35,36]. Nevertheless, the biological and pharmacological effects of CO across the esophageal SE, Barrett’s metaplasia, EAC sequence remain largely unexplored.

**Figure 1:**
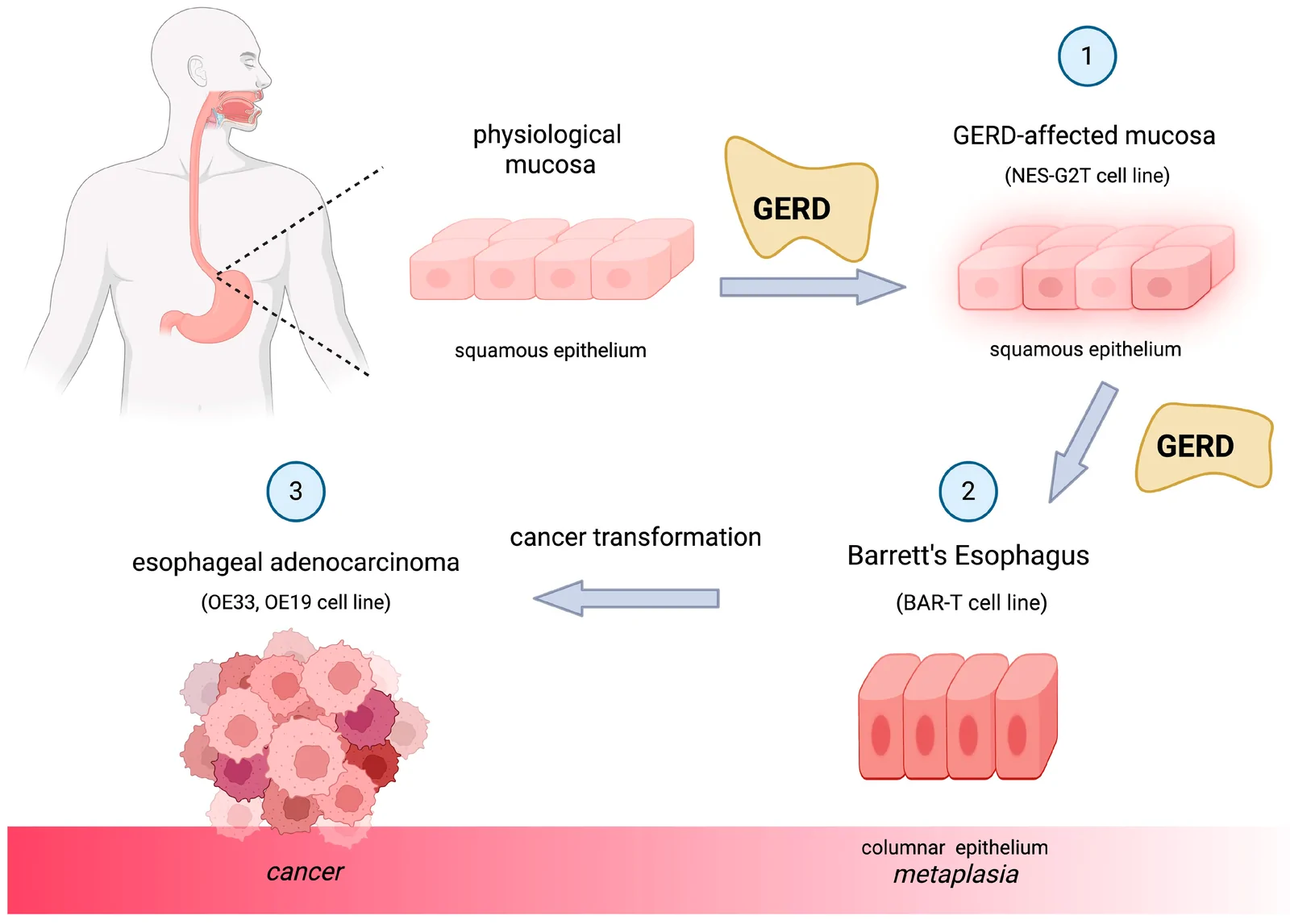
Simplified overview of esophageal adenocarcinoma development and study design. The schematic illustrates progression from gastroesophageal reflux disease (GERD) through Barrett’s esophagus (BE) to esophageal adenocarcinoma (EAC), together with the experimental workflow and cellular models used in the study. Created in BioRender. Magierowski, M. (2026) https://BioRender.com/s3ae5ks.

In our previous works, we proposed and optimized the setting of human esophageal cell line-based in vitro models [37,38] that mimicked the sequence of disease progression and cancer development. Moreover, we demonstrated their clinical relevance by reflecting a BE-specific gene expression profile but without an evaluation of any pharmacological intervention. To address this knowledge gap, we investigated here whether the exogenous delivery of CO via sodium boranocarbonate (CORM-A1) differentially affects esophageal epithelial cells across the squamous epithelium, metaplasia, or adenocarcinoma sequence. CORM-A1 is a water-soluble and widely used CO donor [39,40], which we employed to assess the effects of increased CO bioavailability on cellular metabolic activity and apoptosis/necrosis across subsequent stages of disease progression represented by human GERD-derived squamous epithelial cells (NES-G2T) [41], non-dysplastic Barrett’s epithelial cells (BAR-T) [42,43,44], and the EAC cell lines OE33 and OE19 [45,46], representing poorly differentiated and moderately differentiated adenocarcinoma phenotypes, respectively (Figure 1). We hypothesized that increased CO bioavailability exerts disease-stage-specific and cancer-selective effects during esophageal adenocarcinoma progression. Accordingly, we aimed to determine how CO-releasing CORM-A1 influences cellular metabolic activity, the apoptosis–necrosis balance, and transcriptomic reprogramming across premalignant and malignant stages of esophageal disease progression.

## 2. Materials and Methods

All the cell culture reagents were purchased from Sigma-Aldrich, St. Louis, MO, USA unless otherwise noted.

### 2.1. Cell Cultures

For in vitro experiments, we implemented well established human-derived esophageal cell lines, immortalized with human telomerase reverse transcriptase (hTERT) [45]. The NES-G2T; a squamous esophageal epithelial cell line derived from a GERD patient without BE [41], was a generous gift from Prof. Rhonda F. Souza (Baylor Scott & White Research Institute, Dallas, TX, USA). NES-G2T cells were cultured in 3:1 DMEM (Gibco, Paisley, UK)/F-12 (Gibco, Grand Island, NY, USA) supplemented with 1% HyClone Cosmic Calf Serum (Cytiva, Marlborough, MA, USA), 10 IU/mL penicillin and 10 µg/mL streptomycin, 400 ng/mL hydrocortisone, 20 ng/mL of human recombinant Epidermal Growth Factor (EGF; Gibco, Paisley, UK), 5 µg/mL of transferrin, 5 µg/mL of insulin, 0.1 nM of cholera toxin, 180 µM of adenine, and 0.02 nM 3,3′,5-triiodo-L-thyronine sodium salt.

The BAR-T cell line was established from non-neoplastic, non-dysplastic Barrett’s metaplasia-derived epithelial cells [42,43,44] and kindly gifted by prof. Rhonda F. Souza (Baylor Scott & White Research Institute, Dallas, TX, USA). BAR-T cells were maintained in Keratinocyte Basal Medium 2 (PromoCell, Heidelberg, Germany) supplemented with 5% Fetal Bovine Serum (FBS), 100 IU/mL penicillin and 100 µg/mL streptomycin, 400 ng/mL hydrocortisone, 20 ng/mL of EGF (Gibco, Paisley, UK), 70 µg/mL of Bovine Pituitary Extract (BPE), 5 µg/mL of insulin, 0.1 nM of cholera toxin, 20 µg/mL of adenine and 5 µg/mL of transferrin.

We also used two EAC cell lines: OE33 cell line, derived from an adenocarcinoma of the lower esophagus in stage IIA (UICC) and OE19 cell line, established from an adenocarcinoma of the gastric cardia/esophageal gastric junction in stage III (UICC) [45,46]. The OE33 and OE19 cell lines were a gift from Dr. Ron Smits (Erasmus MC, Rotterdam, The Netherlands). Both cell lines were cultured in RPMI-1640 medium with 5% FBS, 100 IU/mL penicillin and 100 µg/mL streptomycin.

Cells were cultured at 37 °C and 5% CO_2_ in the appropriate medium and grown in a monolayer and then harvested. Cells were detached from T-75 flasks using 0.25% trypsin-EDTA solution (Gibco, Grand Island, NY, USA) for 2–5 min. Cells were centrifuged at 150× *g* for 5 min, and then the supernatant was removed. Each pellet was resuspended in a fresh growth medium. The viability and cell counts were determined using the Invitrogen Countess 3 FL Automated Cell Counter (Thermo Fisher Scientific, Waltham, MA, USA) based on the exclusion of Trypan Blue (Thermo Fisher Scientific, Vilnius, Lithuania) -stained dead cells. Experiments were performed in 3 independent biological replicates.

### 2.2. Preparation of Reagents and Solutions

Prior to each experiment, 1 g of CORM-A1 (sodium boranocarbonate) was weighed and dissolved in phosphate-buffered saline (PBS, Sigma-Aldrich, St. Louis, MO, USA) to prepare a 10 mM stock solution. The stock was filtered through a 0.22 μm membrane filter and immediately diluted in the appropriate fresh culture medium to obtain final concentrations ranging from 0.5 to 500 μM. Fresh stock solutions and aliquots were prepared prior to each use as previously described [39].

iCORM-A1, the CO-depleted inactive form, was generated as described elsewhere with minor modification [39] and used as a negative structural control. Briefly, CORM-A1 was weighed and initially dissolved in 1M HCl and then adjusted by titration with 1 M NaOH to determine pH = 7.4. Distilled H_2_O was added to obtain 10 mM final stock solution. The stock was diluted to proper concentrations ranging from 0.05 μM to 500 μM in the respective cell culture medium. The higher concentrations (≥10 μM) of CORM-A1 and iCORM-A1, which constituted the primary hypothesis-driven dose range, were identical across all cell lines, whereas minor differences in the lowest concentrations reflected technical aspects of independently performed experiments conducted at different time points.

### 2.3. Assessment of Cellular Metabolic Activity (MTT Assay)

Cellular metabolic activity was assessed using the MTT colorimetric assay with thiazolyl blue tetrazolium bromide (MTT; Sigma-Aldrich, Schnelldorf, Germany) [37]. NES-G2T, BAR-T, OE33 and OE19 cells were seeded in 96-well plates (1 × 10^4^ cells/well, 25% confluency) in 3 technical replicates and incubated overnight at 37 °C, 5% CO_2_. Then, CORM-A1 and iCORM-A1 solutions were prepared at appropriate concentrations and applied immediately to the cells for 30 min daily. Controls received medium alone. After incubation, cells were washed with PBS, and fresh growth medium was added (100 μL/well). Untreated cells served as controls. The chemical was applied for 3 consecutive days. After 72 h, 50 μL of the MTT solution was applied to each well and incubated for 4 h at 37 °C. Medium was removed and the formazan product of MTT reduction was dissolved in 75 μL of dimethyl sulfoxide (DMSO; Sigma-Aldrich, St. Louis, MO, USA) per well. Absorbance was measured at 550 nm using the Tecan microplate reader (Sunrise, Männedorf, Switzerland) and expressed as a percentage of metabolic activity relative to untreated control cells. All treatments were performed in three independent experiments.

### 2.4. Cell Survival Analysis by Flow Cytometry

Apoptosis was analyzed using the PE Annexin V Apoptosis Detection Kit I (BD Biosciences, San Jose, CA, USA) according to the manufacturer’s instructions and as previously described [47]. Briefly, NES-G2T, BAR-T, OE33, and OE19 cells (3 × 10^5^ cells/well) were seeded onto 6-well plates and cultured overnight. Cells were subsequently treated with 500 μM CORM-A1 or 500 μM iCORM-A1 for 30 min over three consecutive days. Following each treatment, cells were washed with PBS. After the first and second treatments, fresh complete culture medium was added, and cells were incubated until the next treatment. Untreated cells served as controls.

Immediately after the third treatment and subsequent PBS washing step, approximately 1 × 10^5^ cells were harvested and stained with PE Annexin V for 15 min at room temperature in the dark. Subsequently, 5 μL of propidium iodide and 400 μL of binding buffer were added immediately prior to analysis. Fluorescence intensities of treated samples and controls were analyzed by flow cytometry using a BD FACS Canto™ instrument (Becton Dickinson, New York, NY, USA). Cell populations were classified as viable, early apoptotic, late apoptotic, or necrotic according to Annexin V and propidium iodide staining patterns. Experiments were performed in triplicate.

### 2.5. Cell Cycle Analysis

Cell cycle distribution was analyzed using propidium iodide (PI)/RNase staining (Becton, Dickinson and Company, Franklin Lakes, NJ, USA) followed by flow cytometry. BAR-T, NES-G2T, OE33 and OE19 cells (3 × 10^5^ cells/well) were seeded onto 6-well plates and cultured overnight. Cells were subsequently treated with 500 μM CORM-A1 or 500 μM iCORM-A1 for 30 min over three consecutive days, following the same treatment protocol as described for the apoptosis assay. Following each treatment, cells were washed with PBS. After the first and second treatments, fresh complete culture medium was added, and cells were incubated until the subsequent treatment. Untreated cells served as controls.

Immediately after the third treatment, cells were washed with PBS, harvested, and fixed in cold 70% ethanol. Fixed cells were stored at −20 °C until further analysis. Prior to staining, cells were washed with PBS and resuspended in staining solution containing 100 μg/mL RNase A and 50 μg/mL propidium iodide. Samples were incubated for 15 min at room temperature in the dark and subsequently analyzed using a BD FACSCanto™ flow cytometer (Becton Dickinson, New York, NY, USA). Cell cycle distribution was determined based on cellular DNA content, and the proportions of cells in the G0/G1, S, and G2/M phases were analyzed using ModFit LT 3.0 software.

### 2.6. Experimental In Vitro Model

For BAR-T, OE33, OE19 cell pellets were thawed and cultured in the appropriate medium. Cells were seeded in technical triplicates in 6-well plates (0.3 × 10^6^ cells/well, 25% confluency) and subjected to 3 days of 500 μM CORM-A1 and iCORM-A1 treatment, with a 30 min period (at 37 °C, 5% CO_2_) of exposure per day. When the solution was removed, cells were rinsed with PBS and covered with fresh grown medium. Untreated cells served as controls. Directly after the third treatment, cells were washed with PBS and then lysed for RNA extraction. Total RNA was isolated using the commercially available RNeasy Plus Mini Kit (Qiagen, Hilden, Germany), according to the manufacturer’s protocol. RNA concentration and purity were assessed using the Nano Drop One spectrophotometer (Thermo Fisher Scientific, Waltham, MA, USA). Experiments were run in 3 independent biological replicates.

### 2.7. Analysis of RNA Sequencing Dataset

Sequencing was outsourced to Genomed (Warsaw, Poland). Total RNA isolated from human esophageal epithelial cell lines was subjected to directional RNA sequencing (RNA-seq). Libraries were prepared using the NEBNext^®^ Ultra™ II Directional RNA Library Prep Kit (New England Biolabs, Ipswich, MA, USA) and sequenced on the Illumina NovaSeq X platform (Illumina, San Diego, CA, USA) (paired-end, 2 × 150 bp). A total of 18 samples representing six experimental groups (*n* = 3 biological replicates per group) were analyzed (BAR-T, OE33, OE19 cell lines +/− CORM-A1). Raw sequencing reads were quality-filtered and adapter-trimmed using Cutadapt, followed by quality assessment with FastQC. Reads were aligned to the human reference genome GRCh38 (GCF_000001405.40) using HISAT2 with strand-specific settings (RF). Gene-level read counts were generated using HTSeq based on GFF genome annotations. Differential gene expression analysis was performed in R using the DESeq2 package. Expression changes were modeled using a negative binomial distribution, and statistical significance was assessed with the Wald test. *p* values were adjusted for multiple testing using the Benjamini–Hochberg procedure, and genes with a false discovery rate (FDR) < 0.05 and an absolute log2 fold change (|log2FC|) ≥ 0.5 were considered significantly differentially expressed for downstream analyses and visualization. Functional enrichment analyses of Gene Ontology (GO) terms and Kyoto Encyclopedia of Genes and Genomes (KEGG) pathways were performed using the topGO and clusterProfiler packages, respectively.

### 2.8. mRNA Expression Determined by Real-Time PCR

Total RNA isolation, reverse transcriptase reaction, and quantitative real-time PCR were performed, and relative mRNA expression was calculated using the 2^−ΔΔCt^ method as we previously described [37]. Briefly, following the manufacturer’s manual for the spin column isolation kit (Universal RNA/miRNA Purification Kit, EURx, Gdansk, Poland), the total RNA was extracted, and then the concentration and purity were assessed by a NanoDrop One spectrophotometer (ThermoFisher Scientific, Waltham, MA, USA). For the reverse transcription reaction, a High-Capacity cDNA Reverse Transcriptase Kit (Applied Biosystems, Foster City, CA, USA) was used. Quantitative real-time PCR was performed using Quant Studio 3 (Applied Biosystems, Foster City, CA, USA). Reactions were prepared in 96-well plates and carried out in technical replicates using 2× TaqMan Fast Advanced Master Mix and 20× TaqMan Gene Expression Assays (Thermo Fisher Scientific, Vilnius, Lithuania). Relative mRNA expression was calculated using the 2^−ΔΔCt^ method; untreated cells served as the calibrator and *GAPDH* as the housekeeping gene. Statistical significance was set at *p* < 0.05.

### 2.9. Statistical Analysis

We used GraphPad Prism 11.0.0 (GraphPad Prism Software LCC. Boston, MA, USA) to perform statistical analysis. The data was normalized and presented as the mean ± SEM. Data were analyzed by one-way ANOVA. Bonferroni’s multiple-comparisons test was used for pairwise comparisons between each tested compound and its matched negative control at the corresponding concentration, whereas Dunnett’s multiple-comparisons test was applied for comparisons with the control group. An unpaired Student’s *t*-test was used for pairwise comparisons between the tested compound and the control. For all statistical analyses, the level of significance was set as *p* < 0.05.

## 3. Results

### 3.1. Dose-Dependent Effect of CO Donor on Metabolic Activity of GERD-, BE-, and EAC-Derived Cell Lines

**Figure 2:**
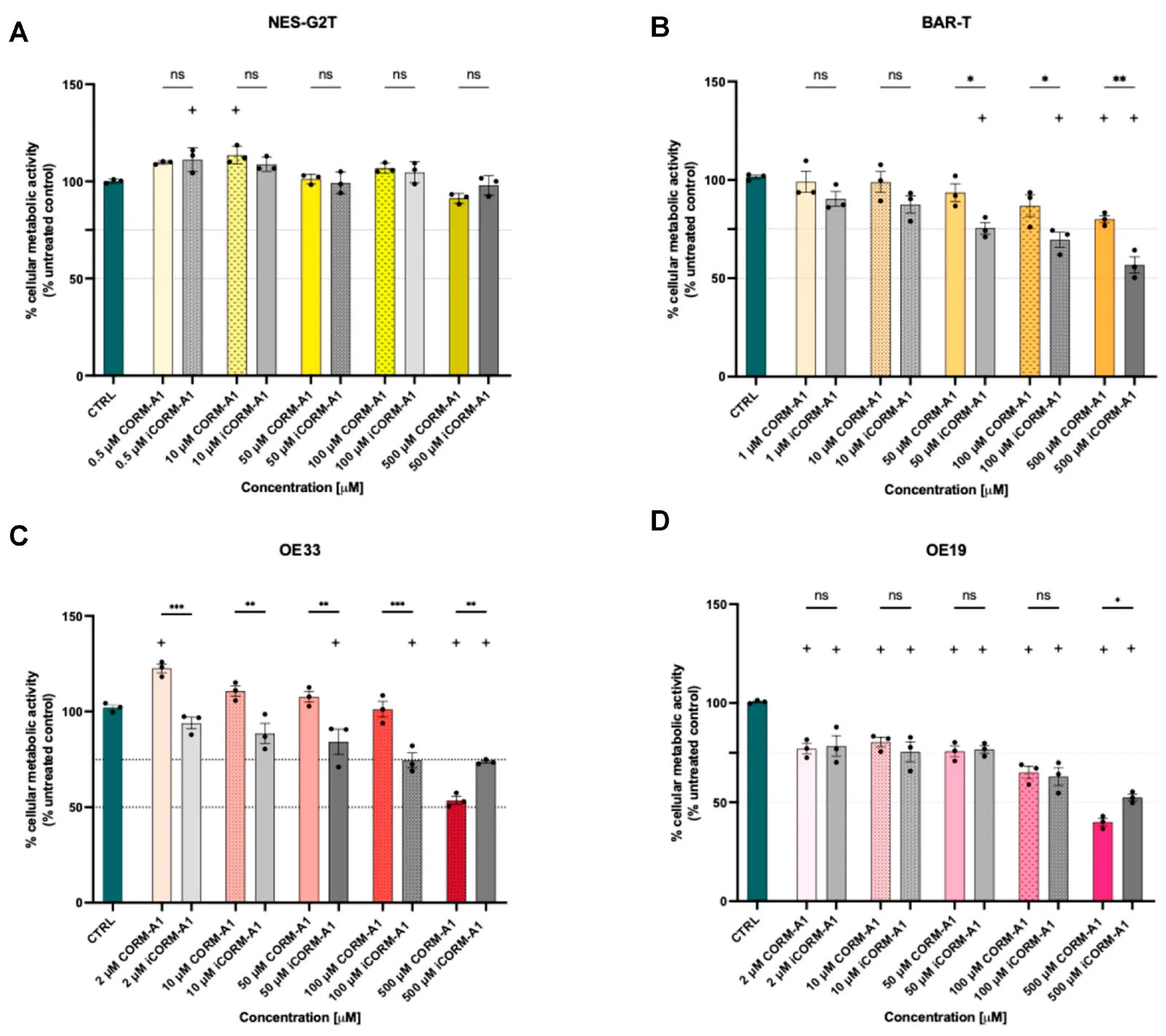
Effect of CORM-A1 and iCORM-A1 on metabolic activity in esophageal cell models. Effects of the CO donor CORM-A1 and its inactive control compound (iCORM-A1) on cellular metabolic activity in in vitro models representing distinct stages of esophageal disease progression: (**A**) NES-G2T, non-transformed esophageal squamous epithelial cells; (**B**) BAR-T, Barrett’s esophagus (BE); and (**C**) OE33 and (**D**) OE19, EAC cells. Cells were exposed to increasing concentrations (0–500 μM) of CORM-A1 or iCORM-A1 for 30 min daily for 3 days; cellular metabolic activity was assessed 72 h later using the MTT assay. Data are presented as the mean ± SEM from three independent experiments and expressed as a percentage of the metabolic activity of untreated control cells. Statistical significance was determined by one-way ANOVA followed by Bonferroni’s multiple-comparisons test for comparisons between CORM-A1 and the corresponding iCORM-A1 treatment groups at the same concentration, or Dunnett’s multiple-comparisons test for comparisons versus untreated controls. Asterisk (*) indicates significant difference versus the corresponding iCORM-A1 group (where: * *p* < 0.05, ** *p* < 0.01, *** *p* < 0.001); cross (+) indicates significant difference versus untreated control, ns-non significant (*p* < 0.05).

To evaluate the effect of CO bioavailability on cellular metabolic activity of esophageal epithelial cell lines, NES-G2T, BAR-T, OE33, and OE19 cells were exposed to increasing concentrations (0–500 μM) of the CO donor, CORM-A1, or its CO-depleted structural control (iCORM-A1) for 30 min, for three consecutive days, followed by MTT assessment. In non-transformed esophageal squamous epithelial NES-G2T cells, neither CORM-A1 nor iCORM-A1 significantly affected cell metabolic activity across the entire concentration range tested (Figure 2A), indicating that transient CO exposure had minimal effects on cellular metabolic activity in non-transformed esophageal epithelial cells.

In contrast, BAR-T, OE33, and OE19 cells exhibited a progressive reduction in metabolic activity following treatment with increasing concentrations of CORM-A1 or iCORM-A1 (Figure 2B–D). In BAR-T cells, which represent a metaplastic yet non-malignant esophageal phenotype and reflect an adaptive response to chronic GERD, iCORM-A1 induced a significant decrease in metabolic activity already at 50 μM, with the effect becoming more significant at higher concentrations and exceeding a 25% reduction at concentrations ≥ 100 μM. In comparison, CORM-A1 exerted a markedly weaker effect, reaching statistical significance only at 500 μM and reducing metabolic activity by less than 25% even at the highest concentration tested (Figure 2B). Therefore, although statistically significant, the CORM-A1-induced reduction in MTT-derived metabolic activity in BAR-T cells remained below 25%, with metabolic activity remaining above 75% of the untreated control even at the highest concentration tested.

In contrast to metaplastic BAR-T cells, EAC-derived cell lines OE33 and OE19 displayed substantially greater reductions in metabolic activity following CORM-A1 treatment. The treatment reduced metabolic activity to below 75% of the untreated control in both cancer cell lines, representing both a statistically and biologically meaningful reduction in the MTT signal (Figure 2C,D). Importantly, at the same concentration, CORM-A1 induced a significantly greater reduction in metabolic activity than the corresponding concentration of iCORM-A1 in both OE33 and OE19 cells, suggesting that CO release contributed to the observed effect. Among the tested cell lines, OE19 cells appeared the most susceptible to increased CO bioavailability, exhibiting the most significant reduction in metabolic activity following exposure to CORM-A1 (Figure 2D).

Collectively, these findings indicate that transient increases in CO bioavailability exert minimal effects on the metabolic activity of non-transformed esophageal epithelial cells, whereas EAC-derived cells display substantially greater reductions in metabolic activity following CORM-A1 treatment. The significantly stronger effect of CORM-A1 than iCORM-A1 in both OE33 and OE19 cells suggests that CO release contributes to the observed reduction in metabolic activity.

### 3.2. Differential Sensitivity of Esophageal Cell Models to CORM-A1-Induced Apoptosis

**Figure 3:**
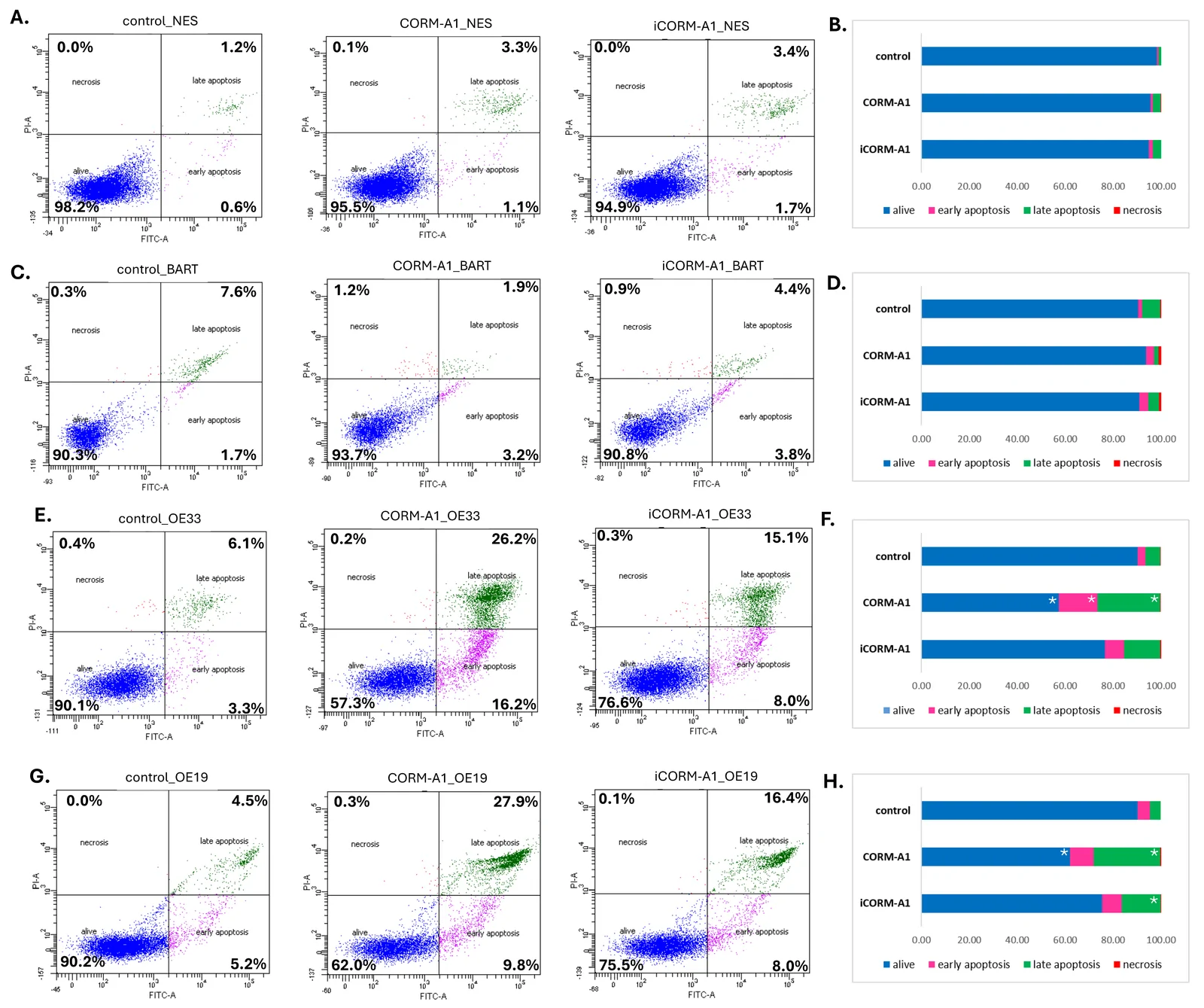
Effect of CORM-A1 and iCORM-A1 on apoptosis in esophageal cell models. Apoptosis was assessed by Annexin V/PI staining followed by flow cytometric analysis after repeated exposure to CORM-A1 or iCORM-A1 (500 μM, 30 min daily for three consecutive days). Representative dot plots and quantitative analyses are shown for NES-G2T (**A**,**B**), BAR-T (**C**,**D**), OE33 (**E**,**F**), and OE19 (**G**,**H**) cells. Cell populations were classified as viable (Annexin V−/PI−), early apoptotic (Annexin V+/PI−), late apoptotic (Annexin V+/PI+), and necrotic (Annexin V−/PI+). The dot plots show representative results from a single experiment, whereas the bar graphs present quantitative data expressed as the mean from three independent experiments (*n* = 3). * *p* < 0.05 versus control.

A flow cytometric analysis revealed a cell line-dependent response to repeated short-term (30 min daily, for three consecutive days) exposure to CORM-A1 (Figure 3). NES-G2T cells were largely resistant to both CORM-A1 and iCORM-A1 treatment, showing only minor changes in the proportions of viable and apoptotic cells compared with untreated controls. Similarly, BAR-T cells did not exhibit an increased apoptotic response following either CORM-A1 or iCORM-A1 exposure.

In contrast, CORM-A1 treatment markedly affected cell survival in both EAC cell lines, OE33 and OE19. In these cells, CORM-A1 reduced the proportion of viable cells and increased the apoptotic populations, with a particularly noticeable increase in late apoptosis. Treatment with iCORM-A1 also affected OE33 and OE19 cell survival, although the observed changes were generally less pronounced than those induced by CORM-A1.

Overall, these results demonstrate a differential response to CORM-A1 across the investigated esophageal cell models. While NES-G2T and BAR-T cells showed limited changes in apoptosis under the applied experimental conditions (Figure 3A–D), OE33 and OE19 cells exhibited a distinct apoptotic response to CORM-A1 (Figure 3E–H). The weaker response to iCORM-A1 in the EAC cell lines suggests that CO release may contribute to the observed effects of CORM-A1.

### 3.3. CORM-A1 Induces Cell Line-Dependent Changes in Cell Cycle Distribution

**Figure 4:**
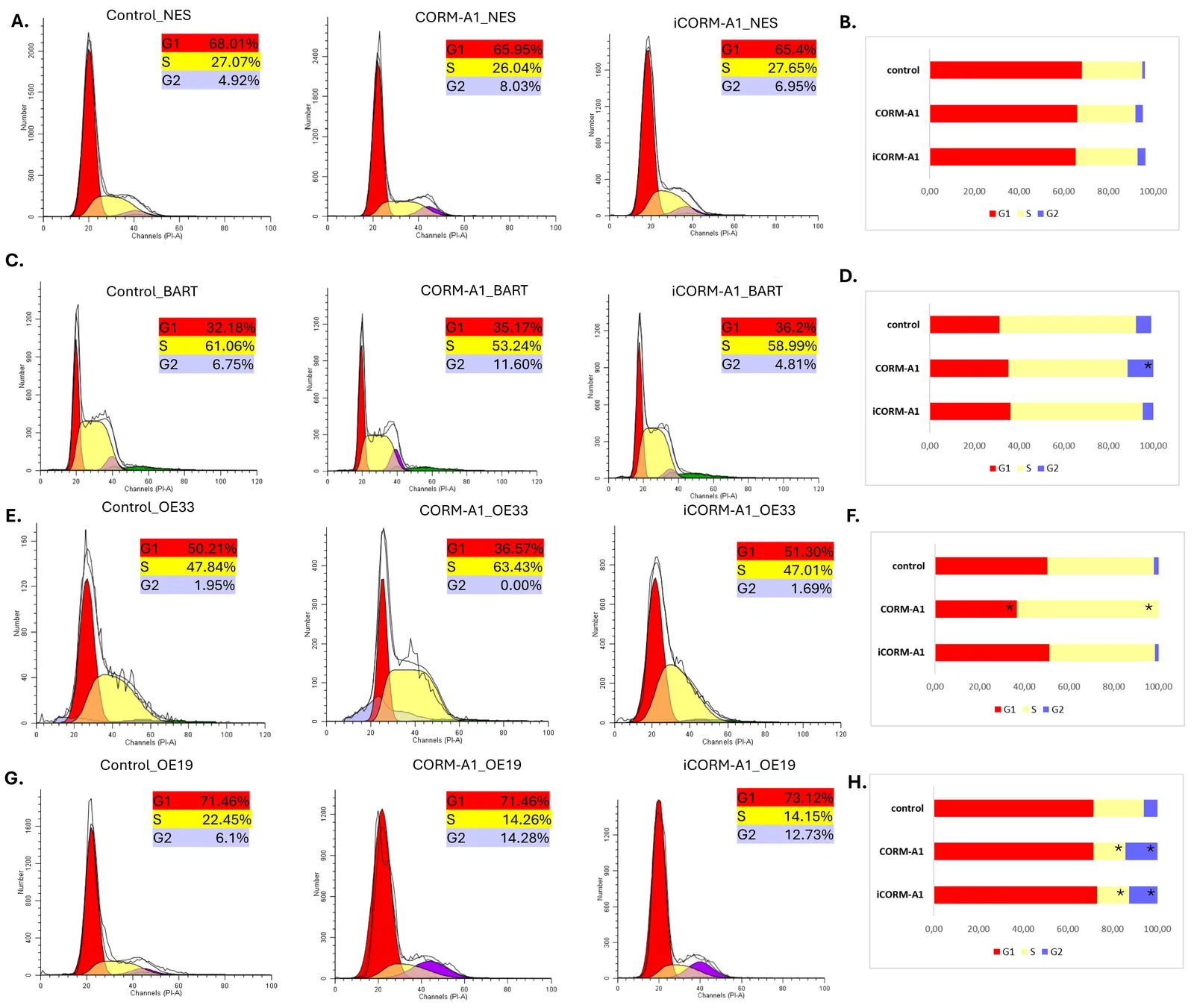
Effect of CORM-A1 and iCORM-A1 on cell cycle distribution in esophageal cell models. Cell cycle distribution was assessed by PI staining followed by flow cytometric analysis after repeated exposure to CORM-A1 or iCORM-A1 (500 μM, 30 min daily for 3 consecutive days). Representative DNA content histograms and quantitative analyses are shown for NES-G2T (**A**,**B**), BAR-T (**C**,**D**), OE33 (**E**,**F**), and OE19 (**G**,**H**) cells. Cells were classified into G0/G1, S, and G2/M phases according to DNA content. The histograms show representative results from a single experiment, whereas the corresponding bar graphs present quantitative data expressed as the mean from three independent experiments (*n* = 3). * *p* < 0.05 versus control.

A cell cycle analysis revealed cell line-dependent changes in response to CORM-A1 and iCORM-A1 treatment (Figure 4). In NES-G2T cells, only minor alterations in cell cycle distribution were observed following either treatment, with the proportions of cells in G1 and S phases remaining largely comparable to those of untreated controls. In BAR-T cells, CORM-A1 treatment was associated with a decrease in the S-phase population and a significant increase in the G2/M fraction, whereas iCORM-A1 induced only minor changes in cell cycle distribution.

More pronounced alterations were observed in the esophageal adenocarcinoma cell lines. In OE33 cells, CORM-A1 treatment resulted in a significant decrease in the G1 population accompanied by a significant increase in the proportion of cells in the S phase, whereas iCORM-A1 had little effect on cell cycle distribution compared with untreated controls. In OE19 cells, both CORM-A1 and iCORM-A1 treatment were associated with a significant reduction in the S-phase population and a significant increase in the G2/M fraction, while the proportion of cells in G1 remained largely unchanged.

Overall, these results indicate that CORM-A1 treatment is associated with cell line-dependent alterations in cell cycle distribution. However, the pattern of these changes differed among the investigated cell models, with no uniform cell cycle response being observed across all cell lines.

### 3.4. Differential Transcriptomic Responses of Barrett’s and Esophageal Adenocarcinoma Cells to CO Treatment

To gain insight into the molecular basis for the markedly greater sensitivity of EAC cells compared with metaplastic Barrett’s epithelial cells to CO treatment, we performed RNA sequencing analysis of BAR-T, OE33, and OE19 cell lines. Cells were exposed to the CO donor CORM-A1 (500 μM) for three consecutive days according to our previously established protocol and published methodology [36,37]. Differential gene expression and pathway enrichment analyses were subsequently performed to identify CORM-A1-responsive molecular programs associated with the metaplastic and neoplastic phenotypes.

**Figure 5:**
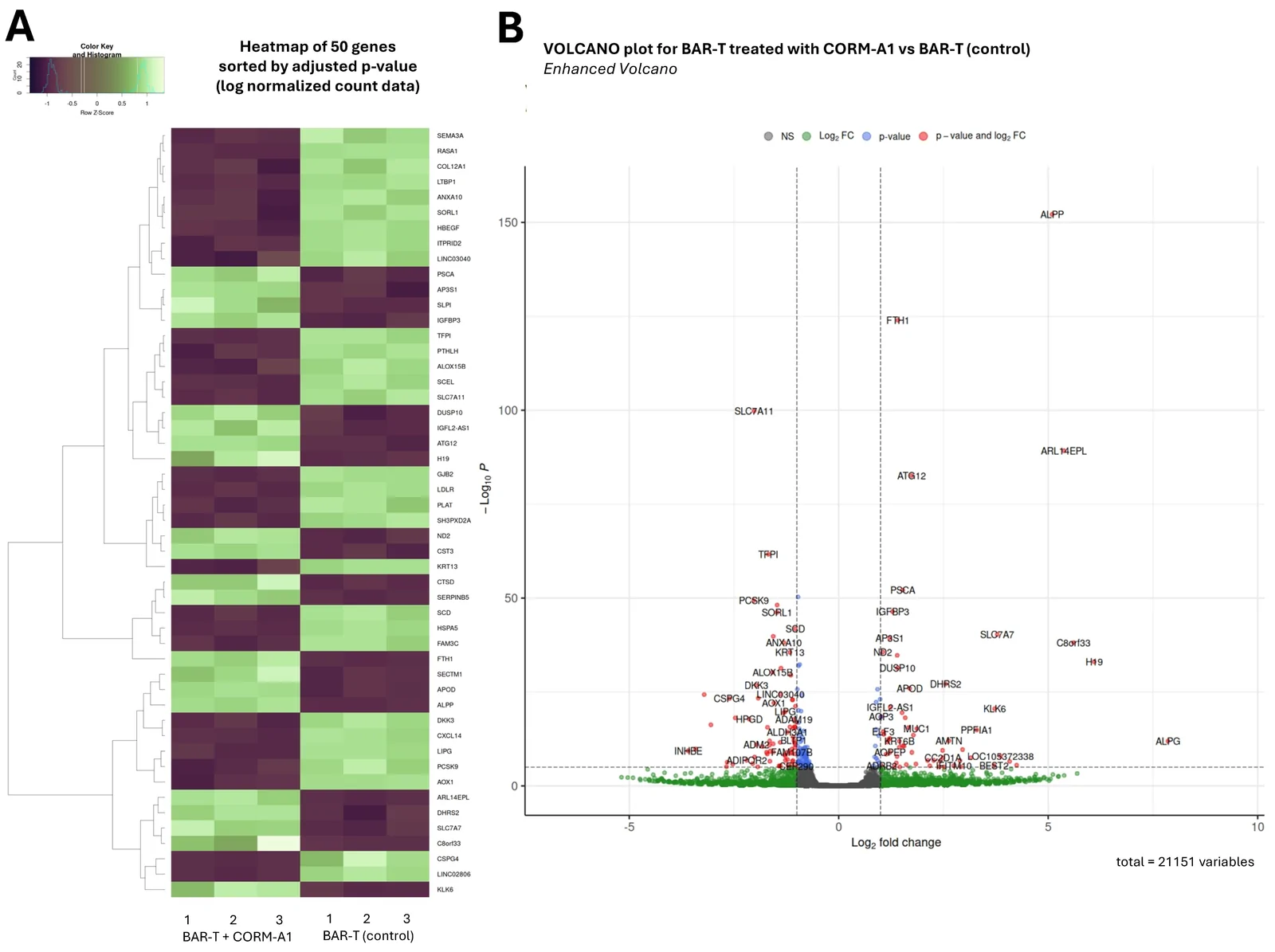
Transcriptomic profiling of BAR-T cells exposed to CORM-A1. (**A**) Heatmap of the top 50 differentially expressed genes identified by RNA sequencing in CORM-A1-treated BAR-T cells relative to untreated controls. Expression values correspond to log-normalized read counts. (**B**) Volcano plot showing differential gene expression between CORM-A1-treated and control BAR-T cells. The dashed vertical lines indicate log2 fold-change thresholds (−0.5 and +0.5). Genes with FDR < 0.05 and |log2FC| ≥ 0.5 were considered significantly differentially expressed. The analysis included 21,151 genes, and selected genes exhibiting the most prominent expression changes are annotated. A total of 418 significantly altered genes were identified, including 139 upregulated and 279 downregulated genes (FDR-adjusted *p* < 0.05). This figure presents representative transcriptomic alterations induced by CORM-A1 treatment in BAR-T cells; the complete RNA-seq dataset and downstream enrichment analyses are provided in Table S1.

Transcriptomic profiling of BAR-T cells revealed a distinct and reproducible transcriptional response, with 418 differentially expressed genes (DEGs) identified among 21,151 analyzed genes (FDR-adjusted *p* < 0.05), including 139 upregulated and 279 downregulated genes (Figure 5B; Table S1). Hierarchical clustering based on the top 50 DEGs demonstrated a clear separation between CORM-A1-treated and control samples, indicating reproducible transcriptional remodeling following CO exposure (Figure 5A).

Among the most strongly induced genes were: *ALPG* (alkaline phosphatase, germ cell), *ALPP* (alkaline phosphatase placental), *H19* (H19 imprinted maternally expressed transcript), *ARL14EPL* (ARF like GTPase 14 effector protein like), *C8orf33* (chromosome 8 open reading frame 33), *SLC7A7* (solute carrier family 7 member 7), and *KLK6* (kallikrein related peptidase 6); whereas *INHBE* (inhibin subunit beta E), *SPDEF* (SAM pointed domain-containing Ets transcription factor), *CSPG4* (chondroitin sulfate proteoglycan 4), *NGFR* (nerve growth factor receptor), *HPGD* (15-hydroxyprostaglandin dehydrogenase), and *CXCL2* (C-X-C motif chemokine ligand 2) ranked among the most prominently downregulated transcripts (Table S1). Functional enrichment analyses revealed a significant overrepresentation of biological processes associated with anatomical structure morphogenesis, the regulation of cell population proliferation, angiogenesis, lipid transport, intermediate filament organization, positive regulation of cell adhesion, and extracellular matrix organization (Table S1). These changes suggest coordinated remodeling of epithelial architecture and tissue homeostasis rather than activation of overt cytotoxic programs.

Consistent with these findings, a GO molecular function analysis demonstrated strong enrichment of cytokine activity, growth factor activity, chemokine activity, extracellular matrix binding, serine-type endopeptidase activity, and serine-type endopeptidase inhibitor activity, indicating modulation of epithelial communication, paracrine signaling, and extracellular matrix remodeling.

GO cellular component analysis further highlighted enrichment of genes associated with the extracellular space, extracellular exosomes, cell surface, apical plasma membrane, plasma membrane, collagen-containing extracellular matrix, basement membrane, and endoplasmic reticulum lumen. Together, these findings point to extensive regulation of epithelial barrier-associated, secretory, and extracellular microenvironmental functions following CO exposure. The enrichment of lipid transport and metabolic regulatory pathways, together with altered expression of multiple cytokine-, growth factor-, and extracellular matrix-related genes, supports a role for CO in promoting adaptive epithelial remodeling and maintenance of tissue homeostasis within Barrett’s epithelium.

Collectively, these findings demonstrate that exogenous CO induces a distinct response in BAR-T cells, characterized by coordinated regulation of epithelial differentiation, tissue remodeling, extracellular signaling, cell adhesion, and metabolic homeostasis, consistent with adaptive and potentially protective transcriptomic reprogramming rather than the cytotoxic response observed in esophageal cancer cells.

**Figure 6:**
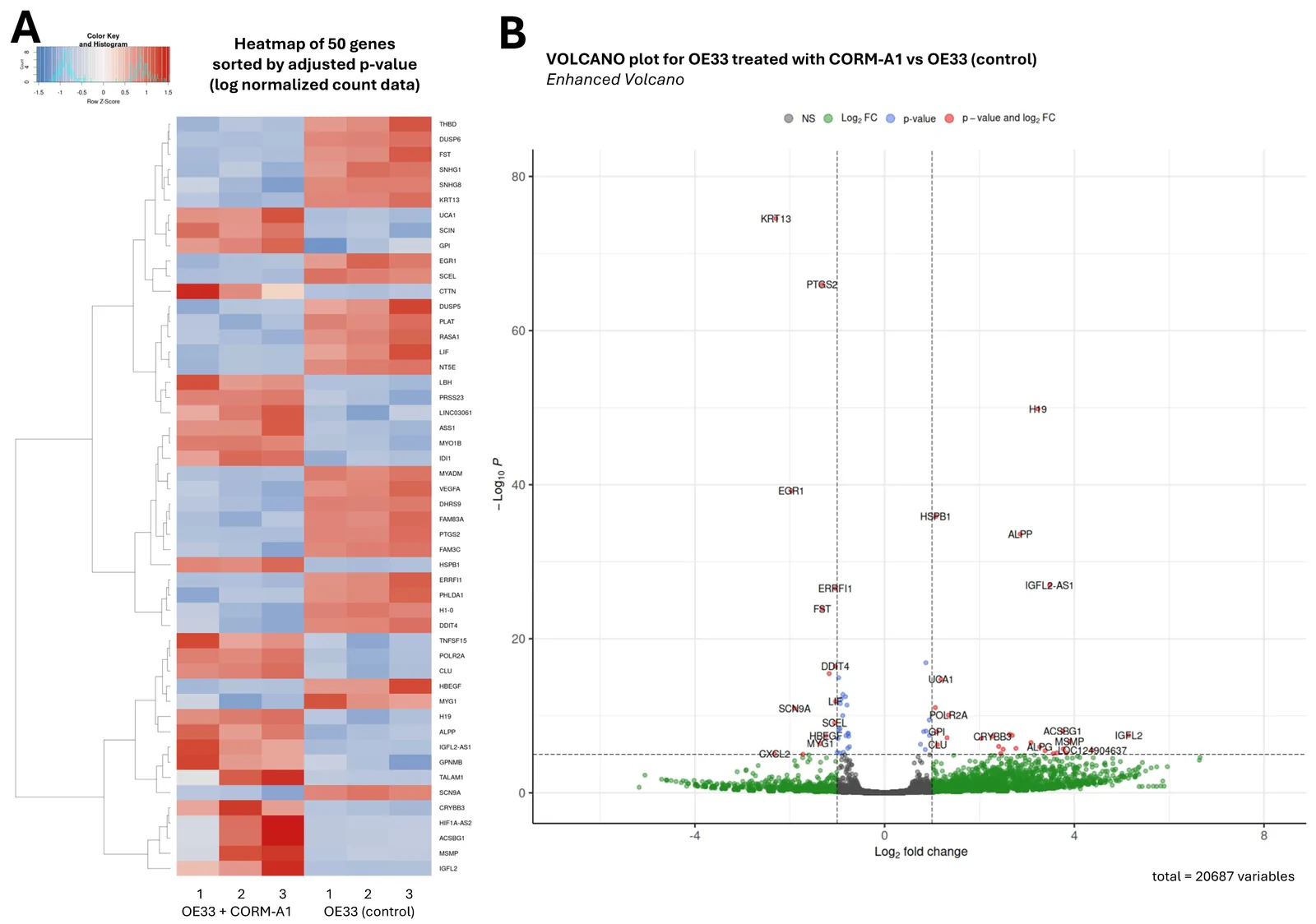
Transcriptomic profiling of OE33 cells exposed to CORM-A1. (**A**) Heatmap of the top 50 differentially expressed genes identified by RNA sequencing in CORM-A1-treated OE33 cells relative to untreated controls. Expression values correspond to log-normalized read counts. (**B**) Volcano plot showing differential gene expression between CORM-A1-treated and control OE33 cells. Dashed vertical lines indicate log2 fold-change thresholds (−0.5 and +0.5). Genes with FDR < 0.05 and |log2FC| ≥ 0.5 were considered significantly differentially expressed. The analysis included 20,687 genes, and selected genes exhibiting the most prominent expression changes are annotated. A total of 220 significantly altered genes were identified, including 161 upregulated and 59 downregulated genes (FDR-adjusted *p* < 0.05). This figure presents representative transcriptomic alterations induced by CORM-A1 treatment in OE33 cells; the complete RNA-seq dataset and downstream enrichment analyses are provided in Table S2.

To investigate whether CO signaling alters gene expression in EAC cells, OE33 cells were exposed to CORM-A1 and subjected to RNA sequencing analysis. Differential expression analysis identified 220 significantly regulated genes (FDR < 0.05) among 20,687 detected transcripts, including 161 upregulated and 59 downregulated genes, indicating a selective but biologically meaningful transcriptional response to CORM-A1 treatment (Figure 6; Table S2). Among the differentially expressed genes, the most strongly induced transcripts included *IGFL2* (IGF like family member 2), *PRADX* (PRC2 and DDX5 associated lncRNA), *H19*, *ALPP*, *IGFL2-AS1* (IGFL2 antisense RNA 1), and *UCA1* (urothelial cancer associated 1), whereas *KRT13* (keratin 13), *PTGS2* (prostaglandin-endoperoxide synthase 2), *EGR1* (early growth response 1), *ERRFI1* (ERBB receptor feedback inhibitor 1), *FST* (follistatin), *DDIT4* (DNA damage inducible transcript 4), and *THBD* (Thrombomodulin) ranked among the most prominently downregulated genes (Table S2). Notably, several of the suppressed transcripts, including *PTGS2*, *EGR1*, *ERRFI1*, *DDIT4* and *VEGFA* (vascular endothelial growth factor A), are implicated in growth factor signaling, stress adaptation, inflammatory responses, angiogenesis, and tumor-microenvironment interactions, indicating that CO exposure preferentially affects pathways associated with cancer cell fitness and adaptation.

Functional enrichment analyses revealed a significant overrepresentation of biological processes related to tissue development, responses to fatty acids, blood coagulation, cellular responses to glucocorticoid stimuli, granulocyte chemotaxis and migration, angiogenesis, and regulation of the ERK1/ERK2 cascade (Table S2). A molecular function analysis further demonstrated enrichment of growth factor activity, cytokine activity, heparin binding, and transmembrane transporter-associated functions, suggesting a preferential modulation of extracellular signaling networks and cell-environment communication. Consistent with these findings, GO cellular component analysis highlighted enrichment of genes associated with the extracellular space, cell surface, cell periphery, extracellular vesicles, membrane-bounded extracellular organelles, and plasma membrane-related compartments, indicating that a substantial proportion of CORM-A1-responsive genes encode proteins functioning at the interface between tumor cells and their surrounding microenvironment. A visualization of the RNA-seq dataset using a volcano plot confirmed the moderate but distinct number of significantly altered transcripts and highlighted a subset of genes exhibiting marked expression changes following CORM-A1 treatment (Figure 6A). Consistently, an unsupervised hierarchical clustering of significantly regulated genes demonstrated a clear separation between control and treated samples, indicating a reproducible transcriptional signature associated with carbon monoxide donor exposure (Figure 6B).

Collectively, these findings indicate that CORM-A1 induces a selective transcriptional response in OE33 cells characterized by coordinated regulation of extracellular signaling, inflammatory mediators, growth factor-associated pathways, angiogenesis-related factors, and stress-response programs. Although the magnitude of transcriptomic remodeling was lower than that observed in non-neoplastic BAR-T cells, the regulated genes included several key modulators of tumor cell adaptation and survival, suggesting a biologically meaningful reprogramming of esophageal adenocarcinoma cells following CO exposure.

**Figure 7:**
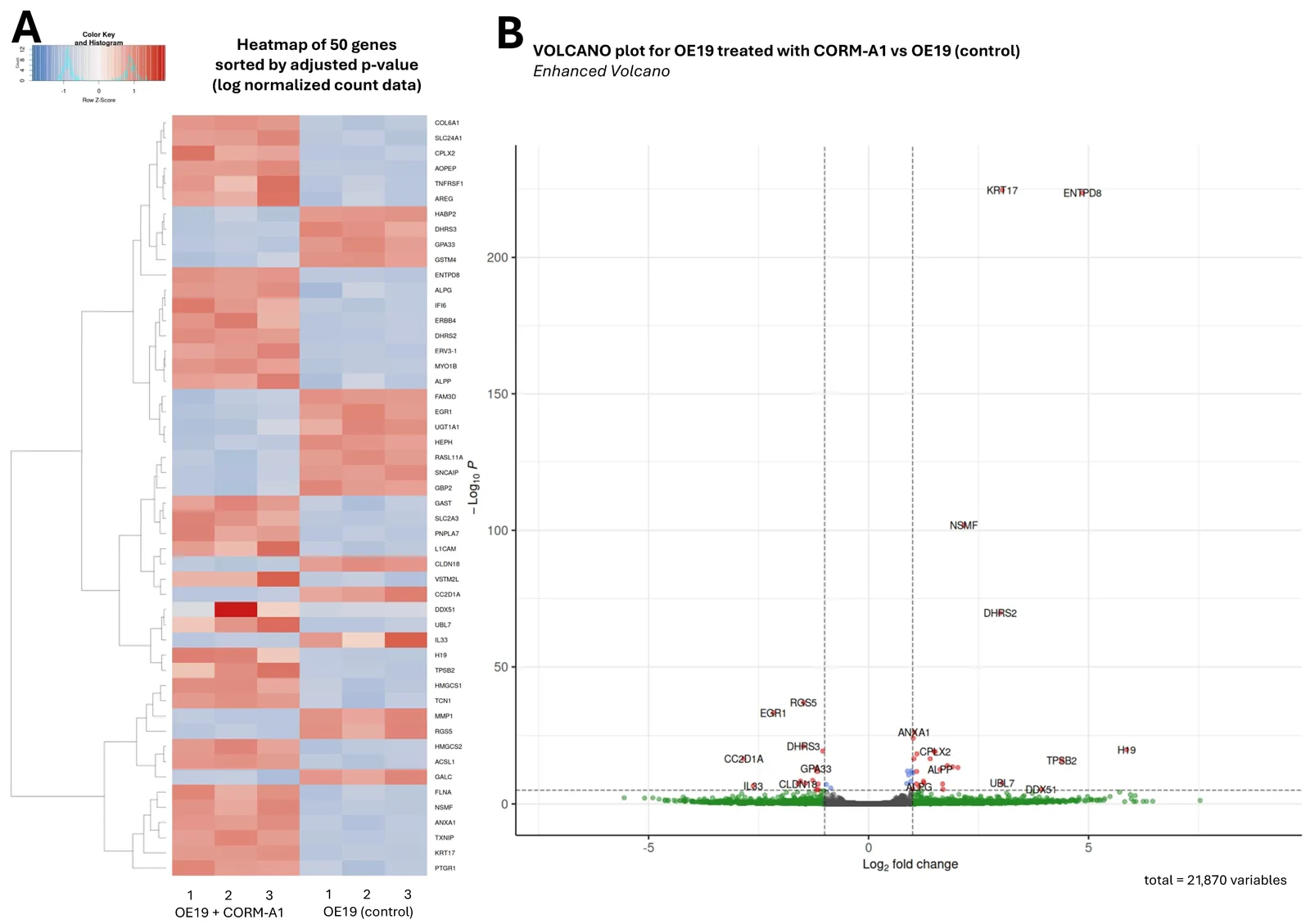
Transcriptomic profiling of OE19 cells exposed to CORM-A1. (**A**) Heatmap of the top 50 differentially expressed genes identified by RNA sequencing in CORM-A1-treated OE19 cells relative to untreated controls. Expression values correspond to log-normalized read counts. (**B**) Volcano plot showing differential gene expression between CORM-A1-treated and control OE19 cells. Dashed vertical lines indicate log2 fold-change thresholds (−0.5 and +0.5). Genes with FDR < 0.05 and |log2FC| ≥ 0.5 were considered significantly differentially expressed. The analysis included 21,870 genes, and selected genes exhibiting the most prominent expression changes are annotated. A total of 64 significantly altered genes were identified, including 40 upregulated and 24 downregulated genes (FDR-adjusted *p* < 0.05). This figure presents representative transcriptomic alterations induced by CORM-A1 treatment in OE19 cells; the complete RNA-seq dataset and downstream enrichment analyses are provided in Table S3.

We also performed bulk transcriptomic profiling of OE19 cells treated with CORM-A1. Differential expression analysis identified 64 significantly regulated genes (FDR < 0.05) among 21,870 detected transcripts, including 40 upregulated and 24 downregulated genes, indicating a relatively modest global transcriptional response to CORM-A1 treatment (Figure 7, Table S3). Hierarchical clustering of the 50 most significantly altered transcripts revealed a clear segregation between treated and control samples, indicating a highly reproducible transcriptional response (Figure 7A). Despite the analysis covering 21,870 detected transcripts, only a limited subset of genes met significance criteria, suggesting that CO exposure induced a focused rather than global remodeling of the OE19 transcriptome (Figure 7B, Table S3).

Among the most strongly upregulated transcripts were *KRT17* (keratin 17), *ENTPD8* (ectonucleoside triphosphate diphosphohydrolase 8), *H19*, *TPSB2* (tryptase beta 2) and

*NSMF* (NMDA receptor synaptonuclear signaling and neuronal migration factor whereas genes including *EGR1*, *DHRS3* (dehydrogenase/reductase 3), *CC2D1A* (coiled-coil and C2 domain-containing 1A), *RGS5* (regulator of G protein signaling 5), and *IL33* (interleukin 33) were markedly downregulated (Figure 7B, Table S3). Notably, several regulated genes, including *EGR1*, *IL33*, *RGS5*, and *FGF19*, are associated with growth factor signaling, inflammatory responses, angiogenesis-related pathways, and cellular adaptation programs, suggesting that CO exposure selectively modulates signaling networks linked to tumor cell fitness and survival. Functional enrichment analyses revealed significant overrepresentation of biological processes related to responses to bacterial and chemical stimuli, regeneration, gland development, estradiol-responsive pathways, glucocorticoid responses, and regulation of programmed cell death (Table S3). Importantly, the enrichment of tyrosine protein kinases receptor family (ERBB) and epidermal growth factor receptor (EGFR) signaling pathways further indicated the selective modulation of growth factor-dependent signaling networks in response to CO exposure. A molecular function analysis demonstrated enrichment of receptor ligand activity, cytokine activity, serine-type endopeptidase activity, and oxidoreductase activity, suggesting preferential regulation of extracellular signaling mediators and stress-response pathways. Consistent with these findings, a GO cellular component analysis highlighted enrichment of genes associated with the extracellular region, collagen-containing extracellular matrix, plasma membrane, cell surface, and secretory vesicle-related compartments, indicating modulation of tumor cell communication with the surrounding microenvironment.

The heatmap analysis confirmed coordinated regulation of these transcripts across biological replicates and demonstrated robust separation of treated and untreated groups (Figure 7A). Together, these findings indicate that increased CO bioavailability induces a distinct but focused transcriptional response in OE19 cells involving a limited number of highly regulated transcripts and pathways associated with extracellular signaling, growth factor responses, inflammatory regulation, programmed cell death, and tumor-microenvironment interactions rather than widespread transcriptomic remodeling. Despite the relatively small number of DEGs, the coordinated suppression of genes such as *EGR1*, *IL33*, and *RGS5*, together with enrichment of ERBB/EGFR-related signaling and programmed cell death-associated pathways, suggests biologically meaningful modulation of cancer cell adaptive responses following CO exposure.

Collectively, transcriptomic profiling demonstrated that CORM-A1 elicited the most pronounced transcriptional response in non-neoplastic BAR-T cells, whereas OE33 and OE19 adenocarcinoma cells exhibited substantially more restricted expression signatures. Despite these quantitative differences, a subset of commonly regulated genes, including *H19* and *ALPP*, emerged across the three cellular models, suggesting the existence of a conserved CORM-A1-responsive transcriptional program superimposed on cell type-specific effects. Notably, whereas CO-induced transcriptional remodeling in BAR-T cells was predominantly associated with epithelial differentiation, tissue homeostasis, and extracellular matrix organization, the responses observed in OE33 and OE19 cells preferentially involved pathways linked to growth factor signaling, cellular adaptation, inflammatory regulation, and tumor-microenvironment interactions.

### 3.5. Comparison of Selected RNA-Seq Target Responses to CORM-A1 and iCORM-A1 by Real-Time PCR

**Figure 8:**
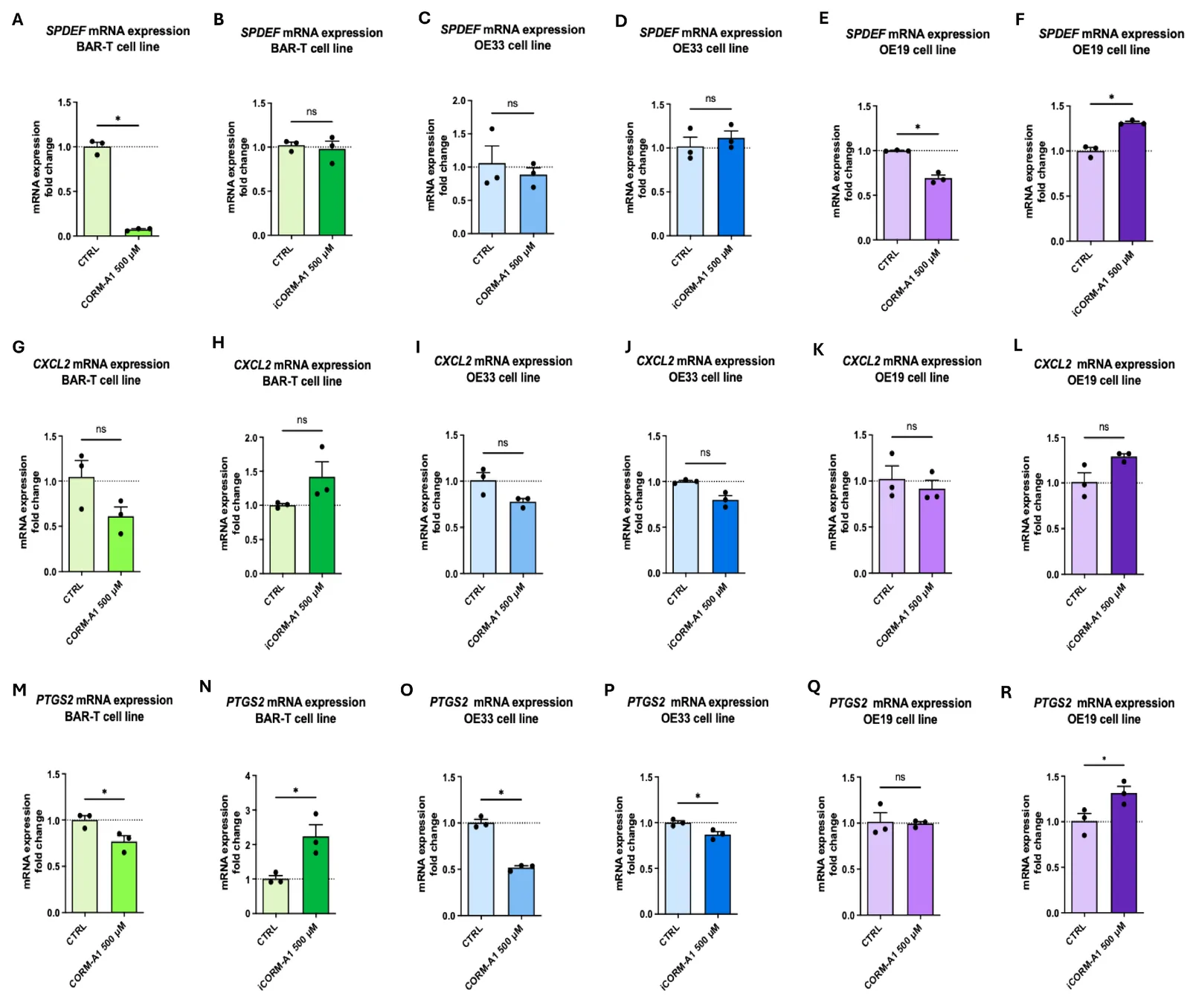
Comparison of selected RNA-seq target responses to CORM-A1 and the inactive control iCORM-A1 by real-time PCR. Expression of *SPDEF* mRNA (**A**–**F**), *CXCL2* mRNA (**G**–**L**), *PTGS2* mRNA (**M**–**R**) in BAR-T, OE33, and OE19 cells following treatment with CORM-A1 or its inactive CO-depleted form (iCORM-A1), 500 μM. Expression levels were normalized to *GAPDH* and were presented as means ± SEMs from three independent biological replicates (*n* = 3). Cells were exposed to the compounds for 30 min daily over three consecutive days. An asterisk (*) indicates a statistically significant difference compared with untreated control cells (*p* < 0.05). ns, not significant.

To further validate the transcriptomic findings and address the specificity of CO-dependent effects, quantitative real-time PCR analyses were performed in BAR-T, OE33 and OE19 cells following repeated exposure to CORM-A1 or its inactive CO-depleted form (iCORM-A1). Cells were treated for 30 min daily over three consecutive days, according to the protocol described above. Expression of two representative RNA-seq targets, *SPDEF*, *CXCL2* and *PTGS2*, is presented in Figure 8.

In BAR-T cells, *SPDEF* (Figure 8A) and *CXCL2* (Figure 8G) are downregulated after CO treatment but *SPDEF* only reached statistical significance, whereas iCORM-A1 shows no significant changes (Figure 8B,H). In OE33 cell line, CO does not affect *SPDEF* (Figure 8C) or *CXCL2* (Figure 8I) gene activity significantly, whereas the lack of CO significantly modulates mRNA expression of *CXCL2.* In OE19 cell line, CO-releasing CORM-A1 downregulates *SPDEF* mRNA levels (Figure 8E), whereas iCORM-A1 upregulates this expression (Figure 8F). In BAR-T cells, *PTGS2* expression was significantly decreased following treatment with the CO donor CORM-A1, whereas iCORM-A1 exerted the opposite effect (Figure 8M,N). In OE33 cells, both CORM-A1 and iCORM-A1 reduced *PTGS2* expression; however, only iCORM-A1 reached statistical significance, whereas CORM-A1 induced an approximately two-fold decrease, indicating potential biological relevance (Figure 8O,P). In OE19 cells, CORM-A1 did not significantly affect *PTGS2* expression, whereas iCORM-A1 significantly increased its expression (Figure 8Q,R).

Overall, these validation experiments confirmed selected transcriptomic observations and demonstrated that the molecular responses induced by CORM-A1 differ from those observed following treatment with the inactive compound, supporting the contribution of CO release to the observed transcriptional effects.

## 4. Discussion

The most important finding of the present study is that increased CO bioavailability exerts fundamentally different biological effects across the SE, esophageal metaplasia, and EAC sequence. While non-transformed SE cells remained largely unaffected and Barrett’s cells mounted a broad adaptive transcriptional response, EAC cells exhibited markedly greater reductions in metabolic activity accompanied by increased apoptosis following CORM-A1 treatment. These findings suggest that neoplastic transformation is associated with increased vulnerability to elevated CO bioavailability.

A particularly intriguing observation was that the magnitude of transcriptomic remodeling did not parallel cellular susceptibility to CO. Barrett’s cells exhibited the most extensive transcriptional response while showing only limited changes in apoptosis following CORM-A1 treatment. In contrast, OE33 and OE19 adenocarcinoma cells displayed substantially fewer transcriptional changes despite exhibiting markedly reduced metabolic activity and enhanced apoptosis. This pattern suggests that metaplastic cells preserve the capacity to mount an adaptive transcriptional response to elevated CO levels, whereas malignant cells respond through a more restricted set of pathways associated with cell fitness, stress adaptation, growth factor signaling, and survival.

The selective nature of the CO response was consistently observed across independent experimental readouts. Importantly, the MTT and flow cytometric assays provide complementary but distinct information, as MTT reduction primarily reflects cellular metabolic activity rather than directly quantifying cell viability. The MTT signal can be influenced by treatment-induced changes in cellular metabolism and reducing capacity independently of cell death, which may account for differences between MTT and Annexin V/PI measurements [48]. Accordingly, the reduced MTT signal observed in BAR-T cells should be interpreted primarily as a decrease in metabolic activity, whereas the limited changes detected by Annexin V/PI indicate that this effect was not accompanied by a pronounced increase in apoptotic cell death. In contrast, EAC-derived OE33 and OE19 cells exhibited both reduced metabolic activity and pronounced apoptosis following CORM-A1 treatment, supporting the concept that the biological consequences of CO exposure are determined by cellular context and stage of disease progression. This interpretation is additionally supported by the suppression of PTGS2/COX-2, a well-recognized mediator of inflammatory and tumor-promoting signaling [25,49], together with reduced metabolic activity and increased apoptosis observed in EAC cells following CORM-A1 treatment.

Cell cycle analysis provided additional evidence for the cell type-dependent nature of the response to increased CO bioavailability. NES-G2T cells showed only minor changes in cell cycle distribution, consistent with their preserved metabolic activity and limited apoptotic response. In BAR-T cells, CORM-A1 increased the G2/M fraction, despite the absence of a pronounced apoptotic response, suggesting that modulation of cell cycle progression may occur independently of overt cell death in this premalignant model. In contrast, the two EAC-derived cell lines exhibited distinct patterns of cell cycle redistribution. CORM-A1 increased the S-phase fraction in OE33 cells, whereas in OE19 cells both CORM-A1 and iCORM-A1 reduced the S-phase fraction and increased the G2/M population. Previous studies have demonstrated that CORMs exposure can affect cell cycle progression together with cell metabolic activity and apoptosis, although these effects may depend on the cellular context and properties of the specific CORM-A1 [50]. Indeed, the anti-cancer activities of CO-releasing molecules have been reported to vary substantially among different cellular models [51]. Accordingly, although both EAC cell lines displayed increased susceptibility to CORM-A1-induced apoptosis in the present study, this response was not accompanied by a common pattern of cell cycle alteration. These findings suggest that the cellular response to CORM-A1 is unlikely to involve a single, uniform cell cycle arrest mechanism and further emphasize the cell type-dependent nature of the response to increased CO bioavailability.

The progression to EAC starts with the only known precursor lesion, BE. As GERD and BE develop gradually and the incidence of EAC continues to rise worldwide, effective preventive strategies aimed at reducing the risk of progression are urgently needed. Current recommendations include risk-based BE surveillance [12], endoscopic and non-endoscopic screening tools (e.g., Cytosponge and high-quality gastrointestinal endoscopy), pharmacological or invasive interventions (e.g., proton-pump inhibitors (PPIs), nonsteroidal anti-inflammatory drugs (NSAIDs), aspirin (ASA), endoscopic eradication therapy (EET), and radiofrequency ablation (RFA)), as well as lifestyle modifications [52,53]. A meta-analysis by Singh et al. demonstrated that PPIs were associated with a 71% reduction in the risk of EAC and/or high-grade dysplasia in patients with BE (adjusted OR 0.29, 95% CI 0.12–0.79) [54]. Similarly, the systematic review by Ko et al. [55] reported significant associations between the use of PPIs and statins and reduced risk of malignant progression, further supporting a preventive role for pharmacological intervention in selected BE patients.

Based on a simplified model of BE development and EAC progression, we investigated the effects of increased CO bioavailability in esophageal epithelial and cancer cell lines. To date, the role of CO in the metaplasia-to-adenocarcinoma sequence remains poorly understood. Previous work in the human bronchial epithelial cell line BEAS-2B demonstrated that CO protects airway epithelial cells against oxidative stress-induced senescence [56]. However, whether similar protective or adaptive effects occur in esophageal epithelial cells has remained unknown.

Numerous studies have investigated the biological effects of CO [57,58,59]. The HO-1/CO pathway and CO activity in neoplastic cells have been extensively studied [59,60,61,62,63], and both pro- and anti-tumorigenic effects, particularly in GI cancers, have been described [29]. While the role of CO in cancer progression remains controversial, our findings provide evidence that increased CO bioavailability may exert anti-tumorigenic effects in esophageal cancer cells.

The implementation of the two distinct esophageal cancer cell lines, OE33 and OE19, in the present study was intended to account for the biological heterogeneity of EAC, representing different stages of tumor progression and levels of aggressiveness. The two cell lines differ in the clinical and histopathological characteristics of the tumors from which they were established, thereby providing complementary models of EAC [45,46]. The OE33 cell line was derived from a poorly differentiated (G3) EAC and classified as stage IIA, whereas OE19 originated from an EAC of the esophagogastric junction/gastric cardia representing stage III disease, with moderate differentiation (G2). This distinction is particularly relevant considering the molecular differences observed between the two models. The relatively limited number of differentially expressed genes identified in OE19 cells may reflect the distinct molecular characteristics of this more advanced tumor model and further emphasizes the importance of using cell lines representing different stages of disease progression. Consequently, the use of OE33 and OE19 cell lines provided a broader experimental perspective and reduced the risk of attributing cell-line-specific findings to EAC as a whole. This observation also aligns with the natural biological differences between cancer cells (OE33 and OE19) and Barrett’s epithelial cells (BAR-T), which, despite sharing the same tissue origin, may display markedly different molecular programs and biological behaviors.

According to our transcriptomic analyses, *H19* emerged as one of the few genes consistently regulated across all three cellular models. *H19* is a maternally expressed long non-coding RNA (lncRNA) [64], although its precise biological role remains incompletely understood. Depending on the cellular context, *H19* has been reported to exert both tumor-promoting and tumor-suppressive functions [65]. In esophageal cancer, Huang et al. [66] demonstrated that increased *H19* expression promotes epithelial-to-mesenchymal transition and invasive behavior in both EAC and ESCC tissues. The recurrent regulation of *H19* observed in our study therefore suggests that this lncRNA may represent part of a conserved CORM-A1-responsive transcriptional program operating across different stages of esophageal disease progression. More broadly, these findings further support an emerging role for long non-coding RNAs in the pathogenesis of BE and EAC.

The interpretation of individual CORM-A1-responsive genes requires particular caution because their biological functions are highly context-dependent and cannot be reliably inferred from transcript abundance alone. Importantly, real-time PCR validation demonstrated that selected transcriptomic alterations, including *SPDEF* and *CXCL2* were not reproduced following treatment with the non-CO-releasing control compound (iCORM-A1), supporting the conclusion that these molecular effects were attributable to increased CO bioavailability rather than chemical structure-related effects. In BAR-T cells, CORM-A1 treatment resulted in downregulation of *SPDEF*, a gene frequently associated with epithelial differentiation and tumor-suppressive functions [67,68]. However, *CXCL2* is a mediator implicated in inflammatory signaling and tumor-microenvironment interactions, was simultaneously suppressed [69]. Thus, the transcriptional response did not display a uniformly protumorigenic direction but rather involved concurrent regulation of genes with potentially opposing biological implications. Consistent with this complexity, pathway enrichment analyses revealed coordinated modulation of epithelial differentiation, tissue remodeling, extracellular signaling, cell adhesion, angiogenesis, and extracellular matrix organization rather than selective activation of a single tumor-promoting program. Accordingly, the observed transcriptional profile should not be interpreted as evidence that CO promotes malignant progression in Barrett’s epithelial cells.

GERD arises from the repeated exposure of the esophageal epithelium to refluxed gastrointestinal contents, promoting chronic inflammation, oxidative injury, and the dysregulation of cellular signaling networks. While acid exposure is a key pathogenic factor, cytokine-driven inflammatory responses and immune cell infiltration have likewise been implicated in disease development [9,70,71]. Consistent with these observations, IL-1β-overexpressing transgenic mice exhibit Barrett’s-like metaplastic changes, suggesting that inflammatory pathways play a critical role in the emergence of BE [72]. Against this background, the transcriptional response observed following CORM-A1 treatment is particularly noteworthy. In OE33 cells, although RNA-seq analysis revealed an increased expression of *IGFL2* and *IGFL2-AS1*, transcripts previously associated with tumor-promoting phenotypes in selected cancer types, these changes occurred concurrently with suppression of several genes involved in inflammatory signaling, growth factor responses, angiogenesis, and tumor microenvironment remodeling, including *PTGS2*, *EGR1*, *ERRFI1*, *DDIT4*, *VEGFA*, and *NT5E.*

Particularly noteworthy is the suppression of *PTGS2* (COX-2), a key mediator of inflammatory signaling that has been extensively implicated in BE progression, EAC development, and tumor-promoting microenvironmental remodeling [25,49]. Importantly, real-time PCR analyses further demonstrated that CORM-A1 reduced *PTGS2* expression, whereas the inactive control compound frequently induced an opposite or distinct transcriptional response. These observations provide additional support for the notion that at least part of the observed molecular effects are related to CO release rather than to nonspecific properties of the donor scaffold itself.

Given the established contribution of inflammatory pathways and immune cell recruitment to GERD-associated epithelial injury and Barrett’s pathogenesis, the downregulation of these transcripts may reflect a broader attenuation of protumorigenic and proinflammatory signaling networks. Accordingly, individual gene expression changes should be interpreted within the context of the overall molecular response rather than as isolated indicators of tumor-promoting activity. Importantly, this transcriptomic profile was accompanied by reduced metabolic activity and enhanced apoptotic cell death following CORM-A1 treatment, arguing against increased short-term cellular fitness under the experimental conditions employed. Nevertheless, as proliferation, clonogenicity, migration, invasion, and tumorigenicity were not directly assessed, future studies will be required to determine whether these gene-specific responses influence long-term malignant behavior.

We certainly acknowledge several limitations of the present study. Bauer et al. have questioned the suitability of some CO-releasing molecules (CORMs) as pharmacological tools for studying CO biology, arguing that part of their biological activity may not be exclusively attributable to released CO [73,74]. Moreover, variability in CO release kinetics and concerns regarding the use of inactive CO donor controls have also been reported [74]. These observations highlight the importance of careful experimental design and appropriate interpretation of studies employing CORMs. However, a detailed characterization of CO-release chemistry was beyond the scope of the present study.

Due to its favorable aqueous solubility, slow and well-characterized CO-release profile (half-life approximately 21 min at pH 7.4 and 37 °C), commercial availability, and previously documented CO-dependent biological activity [39], CORM-A1 was selected for the present study. Notably, CORM-A1 and other CORMs (CORM-2, CORM-3, and CORM-401) have been widely employed in experimental studies investigating the physiological and pathological roles of CO [39,75], including several recent reports [76,77,78]. CO is released from CORM-A1 in a slow and sustained manner [39,77]. Previous studies by Motterlini et al. examining the release kinetics and chemical properties of this group of compounds [39,79,80] provide important context regarding the release characteristics and biological relevance of CORM-A1, including concentrations comparable to the 500 μM dose employed in the present study. Although the nominal concentration of CORM-A1 should not be interpreted as an equivalent CO concentration, a direct quantitative comparison between exogenous CO release from CORM-A1 and endogenous CO generation by heme oxygenases remains challenging because tissue CO bioavailability depends on multiple biological and physicochemical factors. Consequently, the biological relevance of the applied donor concentration should be interpreted primarily in relation to the observed differential cellular responses.

Furthermore, alternative donors may possess limitations of their own, such as metal-based structures, solvent-related effects, or very rapid release kinetics [81]. Consequently, while the development of next-generation CO donors remains an important objective, CORM-A1 continues to represent a useful and broadly accepted experimental tool for investigating CO biology. It is important to note that, in our in vitro cancer model, a higher concentration of CORM-A1 (500 μM) exerted anti-cancer effects. These findings suggest that CO release contributed to the stronger anti-neoplastic effects of CORM-A1 in esophageal cancer cell lines, which were more pronounced than those observed following treatment with the CO-depleted structural control, iCORM-A1. Consistent with this interpretation, iCORM-A1 has been used before as a negative control to discriminate biological effects mediated by CO release from those attributable to the chemical scaffold of CORM-A1 itself [39,57,82]. Although iCORM-A1 affected selected cellular and transcriptional readouts, it should be noted that iCORM-A1 was generated using relatively low concentrations of HCl only at the stock-solution preparation stage and was subsequently extensively diluted in the culture medium before application to cells. Therefore, the contribution of HCl itself to the observed biological responses is expected to be negligible. These findings should be interpreted within the recognized limitations of structural control compounds used in studies investigating CO biology.

Panda et al. [83] demonstrated that BAR-T cells do not fully recapitulate the transcriptomic profile of primary BE and instead retain a more squamous-like molecular signature. Nevertheless, BAR-T cells displayed the most extensive transcriptional response to CO exposure while showing only limited apoptotic changes following CORM-A1 treatment, supporting the existence of adaptive transcriptional programs distinct from those operating in adenocarcinoma cells. These observations should be interpreted in the context of the recognized molecular heterogeneity of Barrett’s esophagus, which is unlikely to be fully represented by any single experimental model [84]. Therefore, further validation in advanced in vitro systems and in vivo models is warranted.

However, the use of cell line-based in vitro models also has several limitations. Their simplified nature does not allow for the investigation of interactions with immune cell infiltration, inflammatory responses, or the tumor microenvironment (TME), all of which may substantially affect cancer biology. In EAC, the TME gathers diverse cell populations and modulates a variety of signaling pathways [85]. Therefore, future studies using translational in vitro models like 3D organoid models, would be beneficial for investigating these complex cellular interactions and cancer-related features [86].

To our knowledge, the present study is the first to demonstrate that increased CO bioavailability elicits distinct, stage-dependent biological and transcriptional responses across the esophageal metaplasia-adenocarcinoma sequence. Our findings suggest that non-malignant and premalignant esophageal epithelial cells primarily mount adaptive responses to CO exposure, whereas EAC cells display reduced metabolic activity accompanied by increased apoptosis following CORM-A1 treatment. These observations support further investigation of CORM-A1-responsive pathways as potential therapeutic targets in EAC.

## 5. Conclusions

In conclusion, our findings demonstrate that the biological effects of CO are highly cell type-dependent and vary substantially across the esophageal metaplasia-EAC sequence. While non-transformed SE cells remained largely resistant to increased CO bioavailability, metaplastic Barrett’s cells exhibited only limited apoptotic responses to CORM-A1 despite being sensitive to the inactive donor scaffold in the MTT assay, suggesting preservation of adaptive mechanisms characteristic of a premalignant phenotype. In contrast, EAC cells displayed a markedly different response, with CORM-A1 inducing significantly greater reductions in metabolic activity than the corresponding inactive compound, accompanied by enhanced apoptotic cell death. Cell cycle analysis further revealed cell type-dependent alterations rather than a uniform pattern of cell cycle arrest, indicating that the cellular response to CORM-A1 is not associated with a single common effect on cell cycle progression. These observations suggest that CO release contributes to the pronounced response observed in malignant cells, although CO-independent effects of the donor cannot be excluded. Transcriptomic analyses revealed pronounced but highly cell type-specific CO-dependent transcriptional responses. While Barrett’s cells exhibited the most extensive transcriptomic remodeling, predominantly affecting pathways related to epithelial differentiation, tissue homeostasis, and extracellular matrix organization, OE33 and OE19 EAC cells displayed more restricted transcriptional responses enriched in pathways associated with growth factor signaling, cellular adaptation, inflammatory regulation, and tumor-microenvironment interactions. Collectively, these data indicate that increased CO bioavailability is preferentially tolerated by non-malignant and premalignant esophageal epithelial cells, while EAC cells exhibit greater reductions in metabolic activity accompanied by increased apoptotic cell death, supporting the concept of CO/CORM-A1-responsive pathways as potential therapeutic targets in EAC.

## Data Availability

The raw RNA sequencing data generated in this study have been deposited in the NCBI Gene Expression Omnibus (GEO) database under accession number GSE344307, https://www.ncbi.nlm.nih.gov/geo/query/acc.cgi?acc=GSE344307, accessed on 21 September 2026. Processed RNA-seq analysis results are provided in Supplementary Tables S1–S3. All other experimental data supporting the findings of this study are presented in the manuscript as individual data points. Additional data are available from the corresponding author upon reasonable request.

## Supplementary Materials

The following supporting information can be downloaded at: https://www.mdpi.com/article/10.3390/biom16101453/s1, Table S1: Transcriptomic analysis of BAR-T cells following sodium boranocarbonate (CORM-A1) treatment; Table S2: Transcriptomic analysis of OE33 cells following sodium boranocarbonate (CORM-A1) treatment; Table S3: Transcriptomic analysis of OE19 cells following sodium boranocarbonate (CORM-A1) treatment.

## Abbreviations

The following abbreviations are used in this manuscript:

ALPG	Alkaline Phosphatase, Germ Cell
ALPP	Alkaline Phosphatase Placental
ARL14EPL	ARF like GTPase 14 Effector Protein Like
ASA	Aspirin
BE	Barrett’s Esophagus
BPE	Bovine Pituitary Extract
C8orf33	Chromosome 8 Open Reading Frame 33
CC2D1A	Coiled-coil and C2 Domain-Containing 1A
CO	Carbon Monoxide
CORM-A1	Carbon Monoxide Releasing Molecule A1
CORMs	Carbon Monoxide Releasing Molecules
CXCL2	C-X-C motif Chemokine Ligand 2
DESeq2	Differential Expression Sequencing 2
DDIT4	DNA Damage Inducible Transcript 4
DMEM/F12	Dulbecco’s Modified Eagle Medium/Ham’s F-12
DMSO	Dimethyl Sulfoxide
DHRS3	Dehydrogenase/Reductase 3
EAC	Esophageal Adenocarcinoma
EDTA	Ethylenediaminetetraacetic Acid
EET	Endoscopic Eradication Therapy
EGF	Epidermal Growth Factor
EGFR	Epidermal Growth Factor Receptor
EGR1	Early Growth Response 1
ENTPD8	Ectonucleoside Triphosphate Diphosphohydrolase 8
ERBB	Tyrosine Protein Kinases Receptor Family
ERRFI1	ERBB Receptor Feedback Inhibitor 1
FastQC	Quality Control for High-Throughput Sequencing Data
FBS	Fetal Bovine Serum
FDR	False Discovery Rate
FST	Follistatin
GERD	Gastroesophageal Reflux Disease
GFF	General Feature Format
GIT	Gastrointestinal Tract
GO	Gene Ontology
GRCh38	Genome Reference Consortium Human Genome Build 38
H19	H19 Imprinted Maternally Expressed Transcript
H_2_S	Hydrogen Sulfide
HCl	Hydrochloric Acid
HISAT2	Hierarchical Indexing for Spliced Alignment to Transcripts 2
HMOX	Heme Oxygenase
hTERT	human Telomerase Reverse Transcriptase
HTSeq	High-Throughput Sequencing
iCORM-A1	Inactive Carbon Monoxide Releasing Molecule A1
IL33	Interleukin 33
IGFL2-AS1	IGFL2 Antisense RNA 1
KEGG	Kyoto Encyclopedia of Genes and Genomes
KLK6	Kallikrein Related Peptidase 6
KRT13	Keratin 13
KRT17	Keratin 17
lncRNA	Long Non-Coding RNA
Log2FC	Log2 Fold Change
MTT	Thiazolyl Blue Tetrazolium Bromide
NaOH	Sodium Hydroxide
NSAiDs	Nonsteroidal Anti-Inflammatory Drugs
NSMF	NMDA Receptor Synaptonuclear Signaling and Neuronal Migration Factor
NO	Nitric Oxide
PBS	Phosphate-Buffered Saline
PE	Phycoerythrin
PPIs	Proton-Pump Inhibitors
PRADX	PRC2 and DDX5 Associated lncRNA
RF	Random Forest
RFA	Radiofrequency Ablation
RGS5	Regulator of G Protein Signaling 5
RNA	Ribonucleic Acid
SE	Squamous Epithelium
SIM	Specialized Intestinal Metaplasia
SLC7A7	Solute Carrier Family 7 Member 7
SPDEF	SAM Pointed Domain-containing Ets Transcription Factor
THBD	Thrombomodulin
TPSB2	Tryptase Beta 2
UCA1	Urothelial Cancer Associated 1
UICC	Union for International Cancer Control
VEGFA	Vascular Endothelial Growth Factor A
